# Supplementary material for: The Female Athlete Triad—the impact of running and type of diet on the regularity of the menstrual cycle assessed for recreational runners
Source: PeerJ. 2022 Mar 2;10:e12903. doi: 10.7717/peerj.12903 (PMC8898003; doi:10.7717/peerj.12903)
Supplement: Supplemental Information 2 [file peerj-10-12903-s002.pdf]

KWESTIONARIUSZ

Wiek…………………..Wzrost………………Masa ciała……………………

Od ilu lat trenujesz?

Jakie minimalne dystanse biegasz? [km]

Jakie maksymalne dystanse biegasz? [km]

Minimalnie, ile razy w tygodniu odbywają się Twoje treningi?

Maksymalnie, ile razy w tygodniu odbywają się Twoje treningi?

Minimalnie, ile godzin trwa jeden trening?

Maksymalnie, ile godzin trwa jeden trening?

Napisz, w którym roku wystąpiła u Ciebie pierwsza w życiu miesiączka?

Jaka jest długość Twojego cyklu miesięcznego?

1 - 24 dni i mniej,
2 - 25-31 dni,
3 - więcej niż 31 dni

Ile dni trwa krwawienie miesięczne?

Ile regularnych cykli miałaś przez ostatnie 12 miesięcy? (0 - wszystkie były nieregularne)

Czy Twoja miesiączka jest:

1 - bezbolesna,
2 - bolesna, ale tylko na początku,
3 - bolesna, przez cały czas trwania

Czy trening podejmowany, w czasie trwania miesiączki ma wpływ na stopnień odczuwania bólu?

1 - TAK zmniejsza ból,
2 - TAK zwiększa ból,
3 - NIE
4 - nie trenuję w czasie miesiączki

Jak określisz stopień bólu podczas miesiączki w skali od 1 do 10 (1 = prawie niezauważalne dolegliwości bólowe, 10 = ból nie do zniesienia)

Czy zdarzyło się, że miesiączka nie wystąpiła przez dłuższy okres czasu po okresie regularnych krwawień?

1 – Tak, poniżej 3 miesięcy,
2 – Tak, pomiędzy 3 a 6 miesięcy,
3 – Tak, powyżej 6 miesięcy,
4 - taka sytuacja u mnie nigdy nie wystąpiła

Czy znasz przyczynę wystąpienia u siebie zaburzeń miesiączkowania?
(wpisz……………………………………………………………………………………...……….)

Czy między miesiączkami występują plamienia? TAK/NIE

Czy stosujesz antykoncepcję hormonalną? TAK/NIE

Czy stosujesz jakąś specjalną dietę dla sportowców np. niskokaloryczną, wysokobiałkową, wegetariańską? TAK/NIE

Jeśli tak o jaką? ……………………………………………………………………………………….

Minimalnie, ile litrów wody dziennie wypijasz?

Maksymalnie, ile litrów wody dziennie wypijasz?

Czy wiesz co to jest Triada Sportsmenek? Jeśli tak, proszę napisz…..
