# Supplementary material for: The Female Athlete Triad—the impact of running and type of diet on the regularity of the menstrual cycle assessed for recreational runners
Source: PeerJ. 2022 Mar 2;10:e12903. doi: 10.7717/peerj.12903 (PMC8898003; doi:10.7717/peerj.12903)
Supplement: Supplemental Information 3 [file peerj-10-12903-s003.pdf]

The author's questionaire

Age…………………..Height………………Weight……………………

How long have you been running for?

What is the minimum distance you run? [km]

What is the maximum distance you run? [km]

What is the minimum number of times you run in a week?

What is the maximum number of times you run in a week??

What is the minimum number of hours for one run?

What is the maximum number of hours for one run?

At what age did menstruation begin for you?

How long is your monthly cycle?

1 - 24 days or less,
2 - 25-31 days,
3 - more than 31 days

For how long does your monthly bleeding last?

How many regular cycles have you had in the last 12 months? (0 - all were irregular)

Is your period:

1 - painless,
2 - painful but only at the beginnning,
3 - painful throughout

Does training during menstruation have an effect on the level of pain?

1 - YES - it lessens the pain,
2 - YES - it increases the pain,
3 - NO
4 - I don’t train during my period

How would you describe your period pain on a scale of 1-10 (1 = the pain is almost negligible, 10 = unbearable pain)

Have you ever missed your period for a long time after a period of regular bleeding?

1 – Yes, for less than 3 months,
2 – Yes, for between 3 and 6 months,
3 – Yes, for more than 6 months,
4 - I have never had such a situation

Do you know the cause of your menstrual disorders?

(give details……………………………………………………………………………………..)

Do you experience spotting between menstruation? YES/NO

Do you use hormonal contraception? YES/NO

Do you use a special diet for athletes e.g.. low calorie, high protein, vegetarian? YES/NO

If yes, which diet?…………………………………………………………………….………

What is the minimum number of litres of water you drink in a day?

What is the maximum number of litres of water you drink in a day?

Do you know what Female Athlete Triad is? If YES, please write……………………………..
